# Supplementary material for: In Pursuit of Optimal Quality: Cultivar-Specific Drying Approaches for Medicinal Cannabis
Source: Plants (Basel). 2024 Apr 8;13(7):1049. doi: 10.3390/plants13071049 (PMC11013261; doi:10.3390/plants13071049)
Supplement: Supplementary file 1 [file plants-13-01049-s001.zip › plants-2923046-supplementary.pdf]

Table S1. Analytical parameters of cannabinoids analyzed by HPLC-PDA.

Name[*]	RT[a] (min)	R^2 [b]	LOQ[c] (μg/mL solvent)	LOD[d] (μg/mL solvent)	Concentration range (μ g/mL solvent)	Accuracy (%)	Repeatability (%CV within day)	Repeatability (%CV between days)
CBDVA	2.5	0.999	0.70	0.21	1-50	3%	1.7%	0.8%
CBDA	3.6	0.999	1.21	0.36	1-1000	3%	0.7%	0.1%
CBGA	3.9	0.999	1.55	0.46	1-100	2%	0.6%	0.2%
CBG	4.1	0.999	2.57	0.77	1-100	2%	0.5%	0.2%
CBD	4.4	0.999	2.68	0.80	1-500	10%	0.6%	0.08%
THCVA	5.6	0.999	1.15	0.34	1-50	2%	2.2%	0.9%
CBN	6.4	0.997	1.43	0.43	1-100	5%	0.6%	0.04%
Δ-9-THC	8.2	0.996	3.69	1.11	1-500	8%	1.4%	1.9%
Δ-8-THC	8.5	0.999	3.12	1.05	1-100	10%	0.8%	0.2%
THCA	10.7	0.999	2.52	0.76	1-1000	4%	0.5%	0.4%
CBCA	12.4	0.996	9.50	3.15	5-100	5%	0.8%	0.2%

[a]RT, retention time in minutes.[b]R^2, coefficient of determination. [c]LOQ, limit of quantitation. [d]LOD, limit of detection. [*]CBDVA, cannabidivarinic acid. CBDA, cannabidiolic acid. CBGA, cannabigerolic acid. CBG, cannabigerol. CBD, cannabidiol. THCVA, (-)-Δ9-trans-tetrahydrocannabivarinic acid. CBN, cannabinol. Δ-9-THC, (-)-Δ9-trans-tetrahydrocannabinol. Δ-8-THC, (-)-Δ8-trans-tetrahydrocannabinol. THCA, (-)-Δ9-trans-tetrahydrocannabinolic acid. CBCA, cannabichromenic acid.

Table S2. Analytical parameters of terpenes analyzed by GC/MS.

Name	RT[a] [min]	R^{2b}	LOQ[c] (μ g/mL solvent)	LOD[d] (μ g/mL solvent)	Concentration range (μg/ml solvent)
α-pinene	8.2	0.9993	7.57	0.53	0.5-250
camphene	9.2	0.9995	6.36	0.48	0.5-250
β-pinene	10.6	0.9992	8.39	1.91	0.5-250
β-myrcene	11.5	0.9996	5.45	0.96	0.5-250
δ-3-carene	12.9	0.9999	1.46	0.77	0.5-250
α-terpinene	13.4	0.9994	7.08	2.2	0.5-250
p-cymene	13.8	0.9994	7.23	0.90	0.5-250
d-limonene	14.0	0.9995	6.11	0.44	0.5-250
ocimene	15.3	0.9996	5.98	3.77	0.5-250
γ-terpinene	16.4	0.9996	5.30	0.99	0.5-250
terpinolene	18.0	0.9995	6.25	0.31	0.5-250
linalool	19.6	0.9997	4.65	2.67	0.5-250
(-)-isopulegol	23.1	0.9996	5.32	3.30	0.5-250
geraniol	30.8	0.9988	20.35	11.12	0.5-250
β-caryophyllene	38.9	0.9997	4.91	0.84	0.5-250
α-humulene	40.6	0.9996	5.33	0.94	0.5-250
nerolidol	46.9	0.9957	22.70	13.19	0.5-250
(-)-guaiol	48.7	0.9996	5.72	4.04	0.5-250
(-)-α-bisabolol	54.1	0.9989	9.58	4.75	0.5-250

[a]RT, retention time in minutes. [b]R^2, coefficient of determination. [c]LOQ, limit of quantitation. [d]LOD, limit of detection.
